# An essential role for Wnt/β-catenin signaling in mediating hypertensive heart disease

**DOI:** 10.1038/s41598-018-27064-2

**Published:** 2018-06-12

**Authors:** Yue Zhao, Chunhong Wang, Cong Wang, Xue Hong, Jinhua Miao, Yulin Liao, Lili Zhou, Youhua Liu

**Affiliations:** 1 State Key Laboratory of Organ Failure Research, National Clinical Research Center of Kidney Disease, Division of Nephrology, Nanfang Hospital, Southern Medical University, Guangzhou, China; 2 Division of Cardiology, Nanfang Hospital, Southern Medical University, Guangzhou, China; 3 Department of Pathology, University of Pittsburgh School of Medicine, Pittsburgh, Pennsylvania USA

## Abstract

Activation of the renin-angiotensin system (RAS) is associated with hypertension and heart disease. However, how RAS activation causes cardiac lesions remains elusive. Here we report the involvement of Wnt/β-catenin signaling in this process. In rats with chronic infusion of angiotensin II (Ang II), eight Wnt ligands were induced and β-catenin activated in both cardiomyocytes and cardiac fibroblasts. Blockade of Wnt/β-catenin signaling by small molecule inhibitor ICG-001 restrained Ang II-induced cardiac hypertrophy by normalizing heart size and inhibiting hypertrophic marker genes. ICG-001 also attenuated myocardial fibrosis and inhibited α-smooth muscle actin, fibronectin and collagen I expression. These changes were accompanied by a reduced expression of atrial natriuretic peptide and B-type natriuretic peptide. Interestingly, ICG-001 also lowered blood pressure induced by Ang II. *In vitro*, Ang II induced multiple Wnt ligands and activated β-catenin in rat primary cardiomyocytes and fibroblasts. ICG-001 inhibited myocyte hypertrophy and Snail1, c-Myc and atrial natriuretic peptide expression, and abolished the fibrogenic effect of Ang II in cardiac fibroblasts. Finally, recombinant Wnt3a was sufficient to induce cardiomyocyte injury and fibroblast activation *in vitro*. Taken together, these results illustrate an essential role for Wnt/β-catenin in mediating hypertension, cardiac hypertrophy and myocardial fibrosis. Therefore, blockade of this pathway may be a novel strategy for ameliorating hypertensive heart disease.

## Introduction

Heart failure is becoming a public healthcare burden on a global scale^1,2^. Approximately 38 million people are diagnosed as heart failure. The number of hospitalized patients with cardiovascular diseases is estimated to be more than 1 million each year, of which 80–90% patients ultimately progress to decompensated heart failure, resulting in poor prognosis and high morbidity and mortality^2,3^. The heart often responds with hypertrophic growth, characterized by increasing myocytes size and cardiac wall thickness, in various pathologic conditions, such as hypertension, myocardial infarction, chronic ischemia and valvular disease^4^. Although this growth in heart size may initially be adaptive or compensatory, mounting studies point out that sustained cardiac hypertrophy is an independent risk factor for heart failure. In fact, left ventricle hypertrophy (LVH) is well recognized as the strongest predictor of the poor prognosis in patients with cardiovascular disorders, and many clinical studies have indicated the close association of an improved patient outcome with LVH regression^5,6^.

Cardiac hypertrophy in diseased conditions is generally believed to be maladaptive and usually leads to changes in the contractile apparatus by expressing fetal isoforms of contractile proteins^7^. In addition, pathologic hypertrophy is often accompanied by an increased expression of non-contractile proteins, such as collagens and fibronectin^8,9^. This leads to myocardial fibrosis, diastolic dysfunction, and eventually heart failure. Several signaling pathways have been involved in the regulation of cardiac hypertrophy, including the renin-angiotensin system (RAS)^4,7,10^, and adrenergic signaling^11–13^. Many studies have shown that chronic infusion of angiotensin II (Ang II), the principal effector of RAS activation, is sufficient to drive cardiac hypertrophy and myocardial fibrosis, in addition to causing hypertension^14,15^. However, exactly how Ang II induces cardiac hypertrophy is incompletely understood.

Wnt/β-catenin is an evolutionarily conserved, developmental signal pathway that is essential for embryonic development, injury repair and tissue homeostasis^8,16–19^. Wnt signal is relatively silent in adult heart tissue, but re-activated after a variety of cardiac injuries ranging from acute ischemic insult to chronic pressure overloading^20–24^. Dysregulated Wnt/β-catenin activation might be implicated in the onset and progression of cardiac hypertrophy. For example, deletion of Dvl, an intermediate for Wnt signal transduction cascade, attenuates cardiac hypertrophy through targeting downstream mediators of glycogen synthase kinase-3^24^. Similarly, inhibition of Dvl also reduces adverse remodeling in ischemic heart^25^. Furthermore, Dapper-1-mediated upregulation of Wnt/β-catenin induces cardiomyocytes hypertrophy and impairs left ventricular (LV) function^26^. These observations suggest a potential role for canonic Wnt signaling in regulating cardiac hypertrophy after injury. However, whether Wnt/β-catenin is also involved in regulating myocyte hypertrophy and cardiac fibrosis *in vivo* in hypertensive heart disease remains to be defined.

In this study, using rat model of chronic Ang II infusion, we have carried out a comprehensive analysis of cardiac expression of all Wnt ligands and demonstrated β-catenin activation in both cardiomyocytes and cardiac fibroblasts *in vivo*. We show that blockade of this pathway by small molecule inhibitor abolished Ang II-triggered cardiac hypertrophy, ameliorated myocardial fibrosis and restored cardiac function, as well as lowered blood pressure. Our studies suggest that inhibition of Wnt/β-catenin signaling may be a novel and effective strategy for therapeutic intervention of hypertensive heart disease.

## Results

### Cardiac hypertrophy is associated with Wnt/β-catenin activation

**Figure 1:**
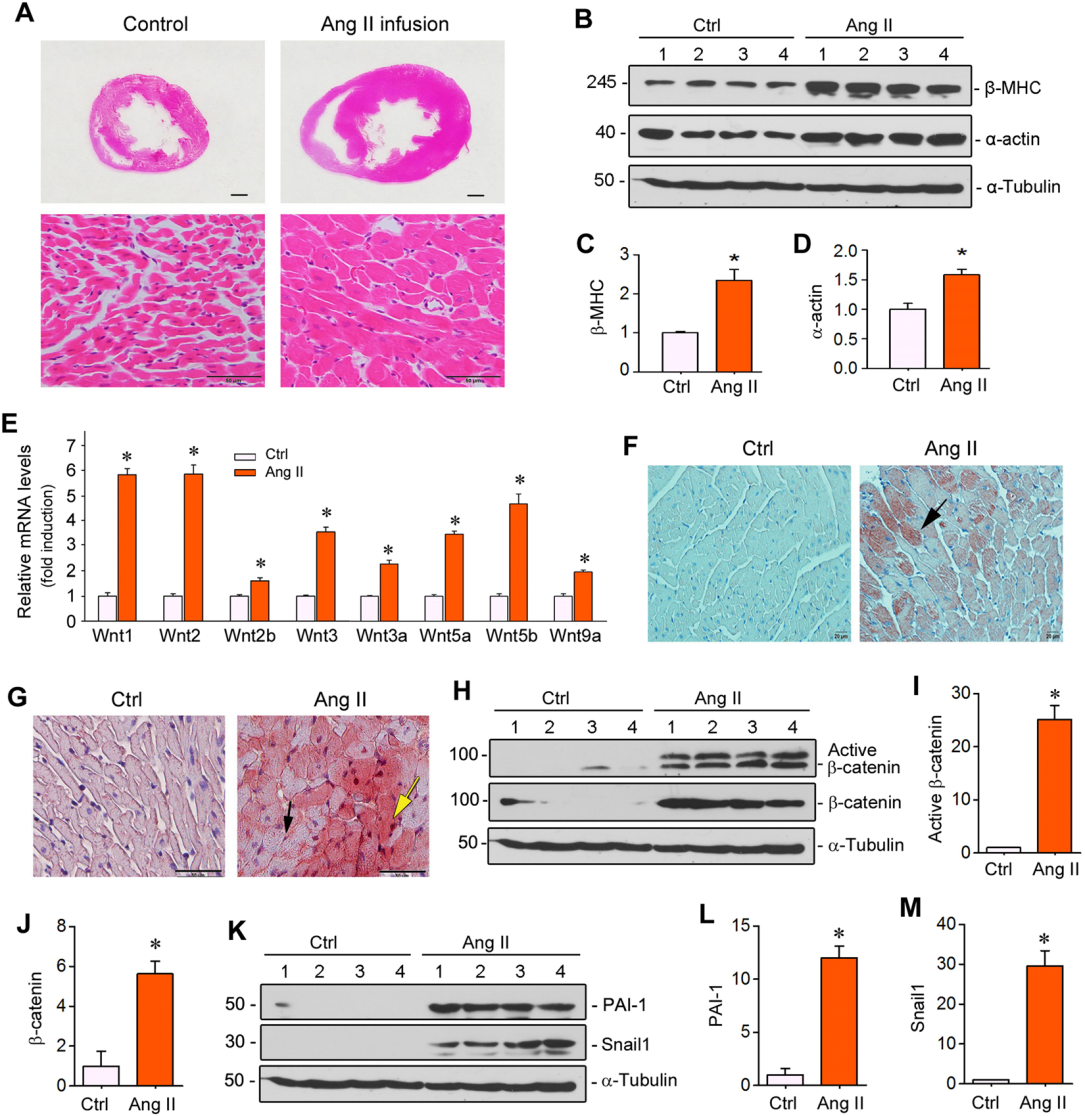
Chronic Ang II infusion induces cardiac hypertrophy and activates Wnt/β-catenin signaling. (**A**) Histological staining (H.E.) revealed overt cardiac hypertrophy in SD rats after 4 weeks of Ang II infusion. Scale bar, 50 µm. (**B**) Western blot analysis showed an increased expression of hypertrophic markers such as β-MHC and α-actin in the heart after Ang II infusion. Whole cardiac lysates were immunoblotted with antibodies against β-MHC, α-actin and α-tubulin, respectively. (**C** and **D**) Quantitative data on β-MHC and α-actin levels in different groups as indicated. *P < 0.05 versus controls (n = 4). (**E**) Quantitative, real-time RT-PCR (qRT-PCR) showed mRNA induction of multiple Wnt ligands in the heart of SD rat after 4 weeks of Ang II infusion. *P < 0.05 versus controls (n = 4). (**F**) Immunohistochemical staining for Wnt3a protein in the heart after Ang II infusion. Arrow indicates positive staining. Scale bar, 20 µm. (**G**) Representative micrographs showed induction of β-catenin protein in the heart after Ang II infusion. Yellow arrow indicates positive cardiomyocyte, whereas black arrow denotes positive cardiac fibroblast. Scale bar, 50 µm. (**H**–**J**) Western blot analysis showed cardiac β-catenin and active β-catenin protein expression in the heart after Ang II infusion. Representative Western blot (**H**) and quantitative data (**I**,**J**) are presented. (**K**–**M**) Western blot analysis showed the protein expression of β-catenin downstream target genes such as PAI-1 and Snail1. Representative Western blot (**K**) and quantitative data (**L**,**M**) are presented. *P < 0.05 versus controls (n = 4). Ctrl, controls. Ang II, angiotensin II.

We utilized rat model of chronic Ang II infusion, which is characterized by hypertension, cardiac hypertrophy and fibrosis^15^, to investigate the mechanism underlying cardiac hypertrophy. As shown in Fig. 1A, cardiac hypertrophy was clearly evident after 4 weeks of Ang II infusion, as the size of heart was significantly increased. Marked increase in myocyte cross-sectional area was also observed (Fig. 1A). Western blotting showed a substantial increase in the protein expression of two hypertrophic biomarkers, β-myosin heavy chain (β-MHC) and α-skeletal muscle actin (α-actin), in the heart of rats at 4 weeks after Ang II infusion (Fig. 1B through D).

To study a possible connection between cardiac hypertrophy and Wnt/β-catenin signaling, we carried out a comprehensive analysis of the mRNA expression of all Wnt ligands in the heart after Ang II infusion. Quantitative, real-time RT-PCR (qRT-PCR) revealed that, of the 19 Wnts tested, eight Wnt ligands were up-regulated by Ang II in the heart, including Wnt1, Wnt2, Wnt2b, Wnt3, Wnt3a, Wnt5a, Wnt5b and Wnt9a (Fig. 1E). Immunohistochemical staining also confirmed the induction of Wnt3a protein, primarily in the hypertrophic cardiomyocytes after Ang II infusion (Fig. 1F). Consistently, β-catenin, the principal intracellular mediator of canonic Wnt signaling, was up-regulated in both cardiomyocyte and interstitial fibroblasts (Fig. 1G). Furthermore, cardiac induction of both active and total β-catenin protein was detected by Western blotting of whole tissue lysates (Fig. 1H through J). Accordingly, PAI-1 and Snail1, two direct downstream targets of Wnt/β-catenin^27,28^, were markedly induced in the heart after Ang II infusion (Fig. 1K through M). These results suggest that Ang II-induced cardiac hypertrophy is associated with Wnt/β-catenin upregulation and activation in rats.

### Inhibition of β-catenin signaling prevents Ang II-induced cardiac hypertrophy

**Figure 2:**
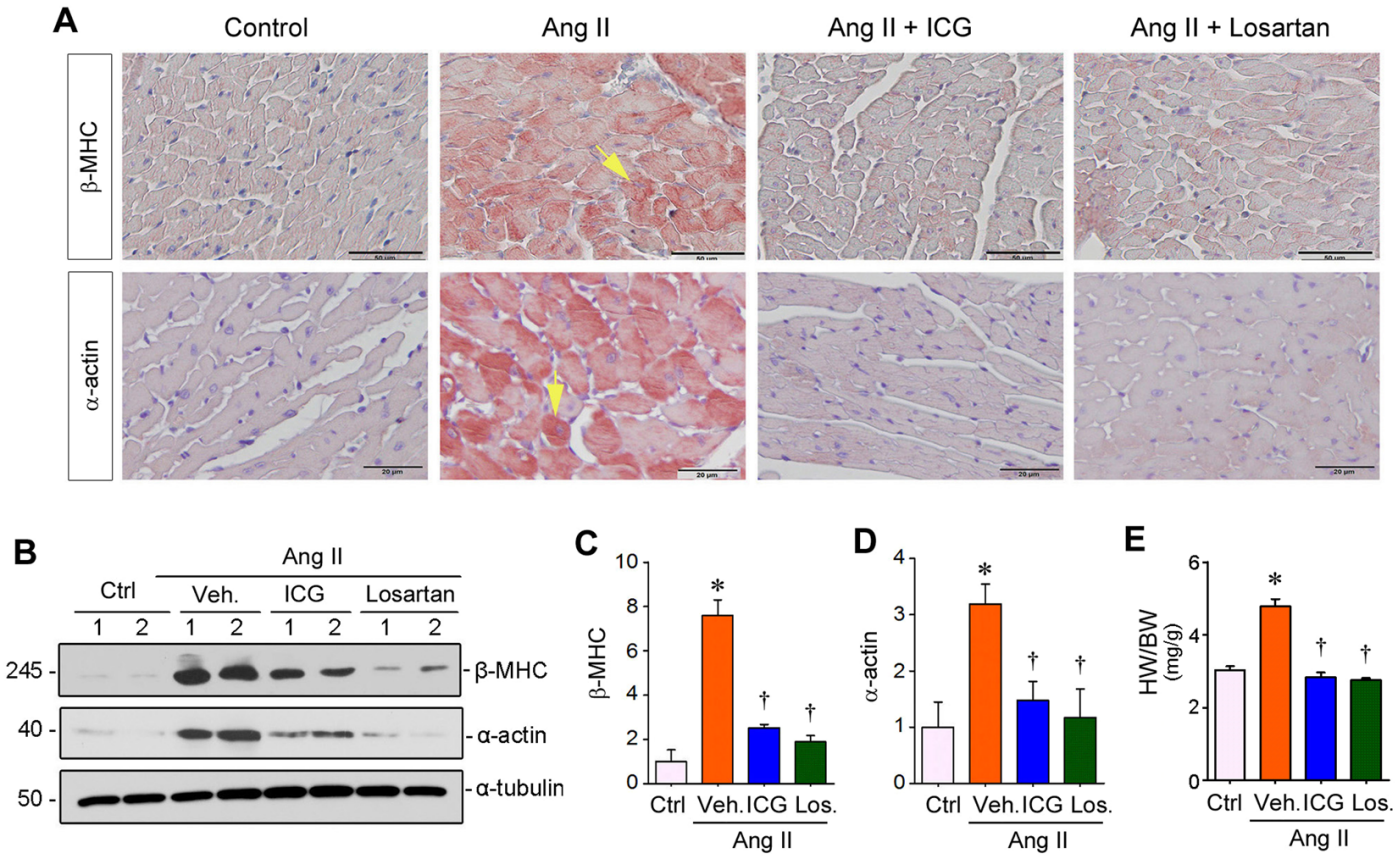
Inhibition of β-catenin signaling ameliorates Ang II-induced cardiac hypertrophy. (**A**) Representative micrographs showed immunohistochemical staining for cardiac β-MHC and α-actin proteins in various groups as indicated. Arrows indicate positive staining. Scale bar, 50 µm (top panel) and 20 µm (bottom panel). (**B**–**D**) Western blot analysis of β-MHC and α-actin in the heart after various treatments as indicated. Representative Western blots (**B**) and quantitative data for β-MHC (**C**) and α-actin (**D**) are presented. *P < 0.05 versus controls; ^†^*P* < 0.05 versus Ang II alone (n = 5). (**E**) Ratio of heart weight/body weight of SD rats in various groups as indicated. HW, heart weight. BW, body weight. Ctrl, sham controls; Veh., vehicle; ICG, ICG-001; Los., losartan. *P < 0.05 versus sham controls; ^†^*P* < 0.05 versus Ang II alone (n = 5).

We next investigated the role of Wnt/β-catenin in mediating cardiac hypertrophy by blocking this pathway. To this end, rats were treated with ICG-001, a small molecule inhibitor that specifically impedes β-catenin-mediated gene transcription^29–31^. As shown in Fig. 2A, ICG-001 almost completely prevented the protein expression of β-MHC and α-actin in the heart after Ang II infusion, as shown by immunohistochemical staining. As expected, injections of losartan, the Ang II receptor type 1 (AT1) blocker, also inhibited Ang II actions and prevented cardiac expression of these hypertrophic makers (Fig. 2A).

We further assessed the expression of hypertrophic markers by using Western blot analysis, a more quantitative approach. As illustrated in Fig. 2B through D, ICG-001 substantially inhibited cardiac β-MHC and α-actin expression. Both β-MHC and α-actin levels in the Ang II-infused rats after ICG-001 treatment were comparable to that in control group. Consistent with these hypertrophic markers, heart/body weight ratio was also significantly increased after Ang II infusion, and ICG-001 and losartan completely normalized heart weight and size in these rats (Fig. 2E). These results indicate a critical role for canonic Wnt/β-catenin signaling in mediating Ang II-induced cardiac hypertrophy.

### Inhibition of β-catenin signaling prevents cardiac geometric abnormalities

**Figure 3:**
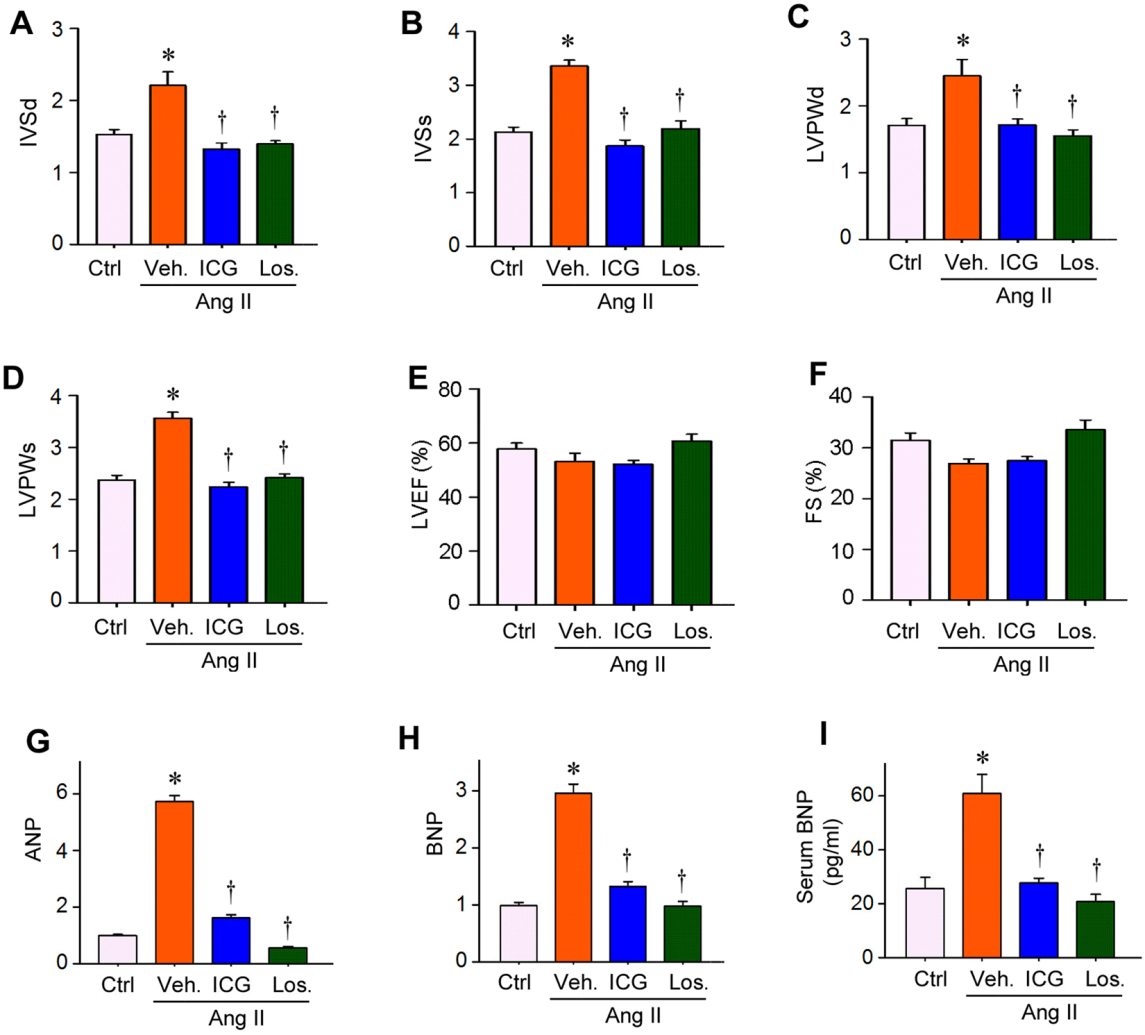
ICG-001 prevents geometric abnormalities and inhibits cardiac injury after Ang II infusion in rats. (**A**–**D**) Echocardiography showed cardiac geometric changes in various groups as indicated. Parameters of cardiac structure measured by echocardiography were as follows, interventricular septum depth at end-diastole (IVSd) (**A**), interventricular septum depth at end-systole (IVSs) (**B**), left ventricular posterior wall depth at end-diastole (LVPWd) (**C**), and left ventricular posterior wall depth at end-systole (LVPWs) (**D**), at 4 weeks after Ang II infusion in SD rats. (**E**,**F**) Echocardiography showed left ventricular ejection fraction (LVEF) and fractional shortening (FS) in rats at 4 weeks after various treatments. (**G**,**H**) qRT-PCR showed the mRNA expression of cardiac ANP and BNP in various groups as indicated. *P < 0.05 versus controls; ^†^*P* < 0.05 versus Ang II alone (n = 5). (**I**) Serum BNP levels in SD rats after various treatments as indicated. Serum BNP protein level was assessed by a specific ELISA. Ctrl, sham controls. Veh, vehicle. ICG, ICG-001. Los, Losartan. *P < 0.05 versus controls; ^†^*P* < 0.05 versus Ang II alone (n = 5).

We further studied cardiac structural abnormalities in rats at 4 weeks after Ang II infusion by using echocardiography. Ang II infusion led to cardiac geometric abnormalities, as reflected by an increased values of interventricular septum depth at end-diastole (IVSd) (Fig. 3A), interventricular septum depth at end-systole (IVSs) (Fig. 3B), left ventricular posterior wall depth at end-diastole (LVPWd) (Fig. 3C), and left ventricular posterior wall depth at end-systole (LVPWs) (Fig. 3D). Both ICG-001 and losartan were equally effective in mitigating these changes (Fig. 3A through D). However, cardiac function such as left ventricular ejection fraction (LVEF) and fractional shortening (FS) were not significantly changed among different groups (Fig. 3E,F), which is typical in the early phase of this Ang II infusion model^32^.

We also assessed cardiac expression of atrial natriuretic peptide (ANP) and B-type natriuretic peptide (BNP), markers for left ventricular dysfunction and cardiac injury. As shown in Fig. 3G,H, ICG-001 and losartan repressed Ang II-induced cardiac mRNA expression of ANP and BNP, as assessed by qRT-PCR. Similarly, serum BNP level was elevated in rats after Ang II infusion, and ICG-001 treatment effectively normalized its level in the circulation (Fig. 3I). Therefore, inhibition of β-catenin by ICG-001 protects against Ang II-triggered geometric abnormalities and cardiac damage.

### Inhibition of β-catenin signaling suppresses Ang II-induced cardiac fibrosis

**Figure 4:**
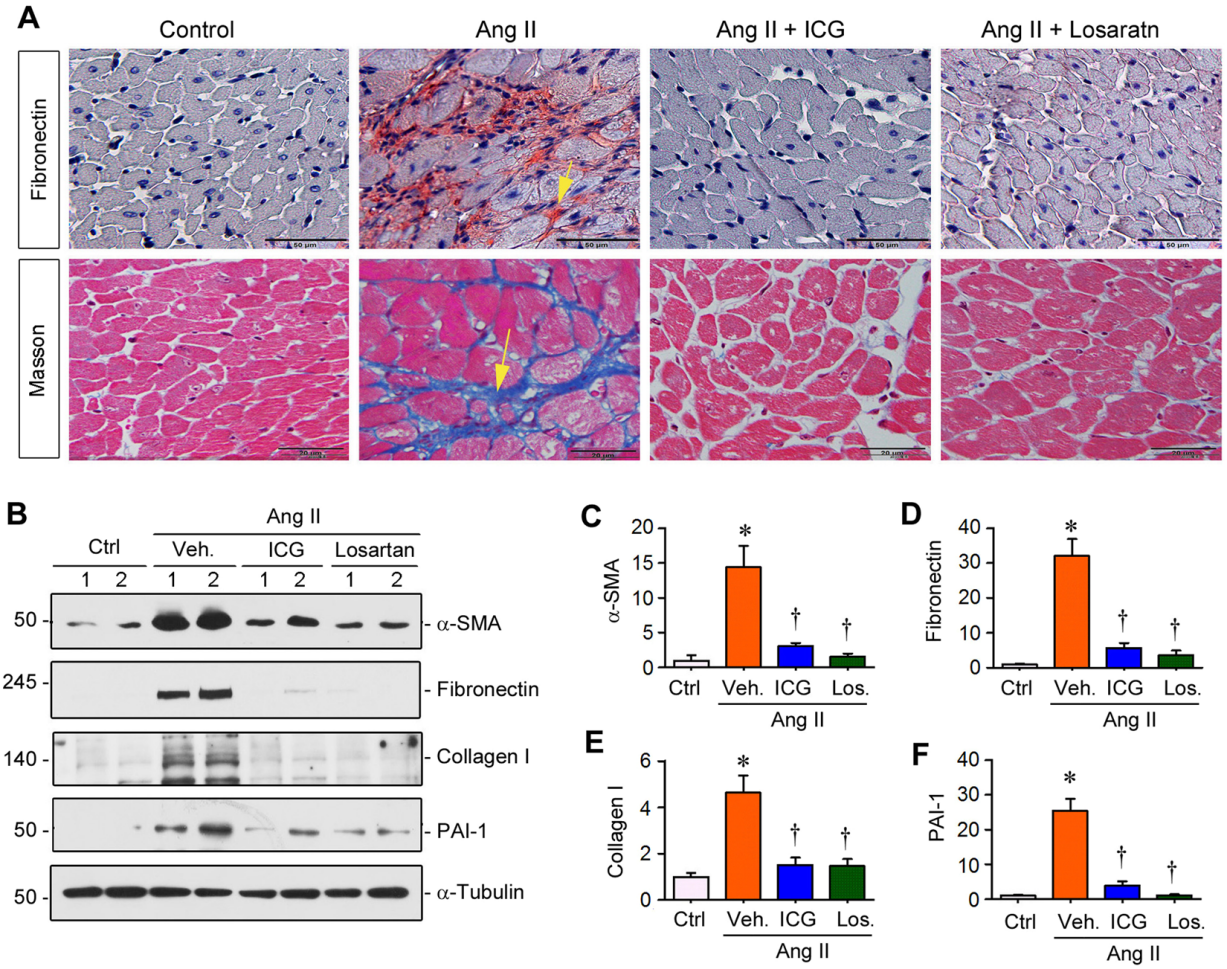
Inhibition of β-catenin signaling by ICG-001 suppresses Ang II-triggered cardiac fibrosis. (**A**) Representative micrographs showed fibronectin protein and collagen deposition in the heart after various treatments as indicated. Immunohistochemical staining for fibronectin protein (upper panel) and Masson’s trichrome staining for collagen deposition (lower panel) are shown. Arrows indicate positive staining. Scale bar, 50 µm (top panel) and 20 µm (bottom panel). (**B**) Representative Western blot showed the protein levels of α-SMA, fibronectin, collagen I and PAI-1 in various groups as indicated. (**C**–**F**) Quantitative data on the protein levels of α-SMA, fibronectin, collagen I and PAI-1 in various groups as indicated. Relative protein levels over the controls (fold induction) are presented. Ctrl, sham controls. ICG, ICG-001. Los, Losartan. *P < 0.05 versus controls; ^†^*P* < 0.05 versus Ang II alone (n = 5).

We then investigated the effect of inhibition of β-catenin signaling by ICG-001 on cardiac fibrosis, characterized by fibroblast activation and matrix accumulation. As shown in Fig. 4A, Ang II infusion caused substantial accumulation of fibronectin in the interstitial compartment of the heart. However, ICG-001 dramatically reduced its accumulation and extracellular deposition. Similarly, Masson’s trichrome staining revealed marked deposition of collagens in cardiac interstitium at 4 weeks after Ang II infusion (Fig. 4A, arrow), and ICG-001 completely mitigated these fibrotic lesions. As a positive control, inhibition of AT1 by losartan abolished cardiac fibrosis as well (Fig. 4A).

We also examined cardiac expression of α-smooth muscle actin (α-SMA), a molecular signature for myofibroblast activation. As shown in Fig. 4B,C, cardiac α-SMA protein was induced after Ang II infusion, and ICG-001 substantially inhibited its expression, suggesting that ICG-001 effectively blocks the activation of myofibroblasts in cardiac fibrogenesis^7,8^. Consistently, ICG-001 also abolished Ang II-induced cardiac fibronectin, collagen I and PAI-1 protein expression (Fig. 4B and D through F).

### Inhibition of β-catenin signaling lowers blood pressure

**Figure 5:**
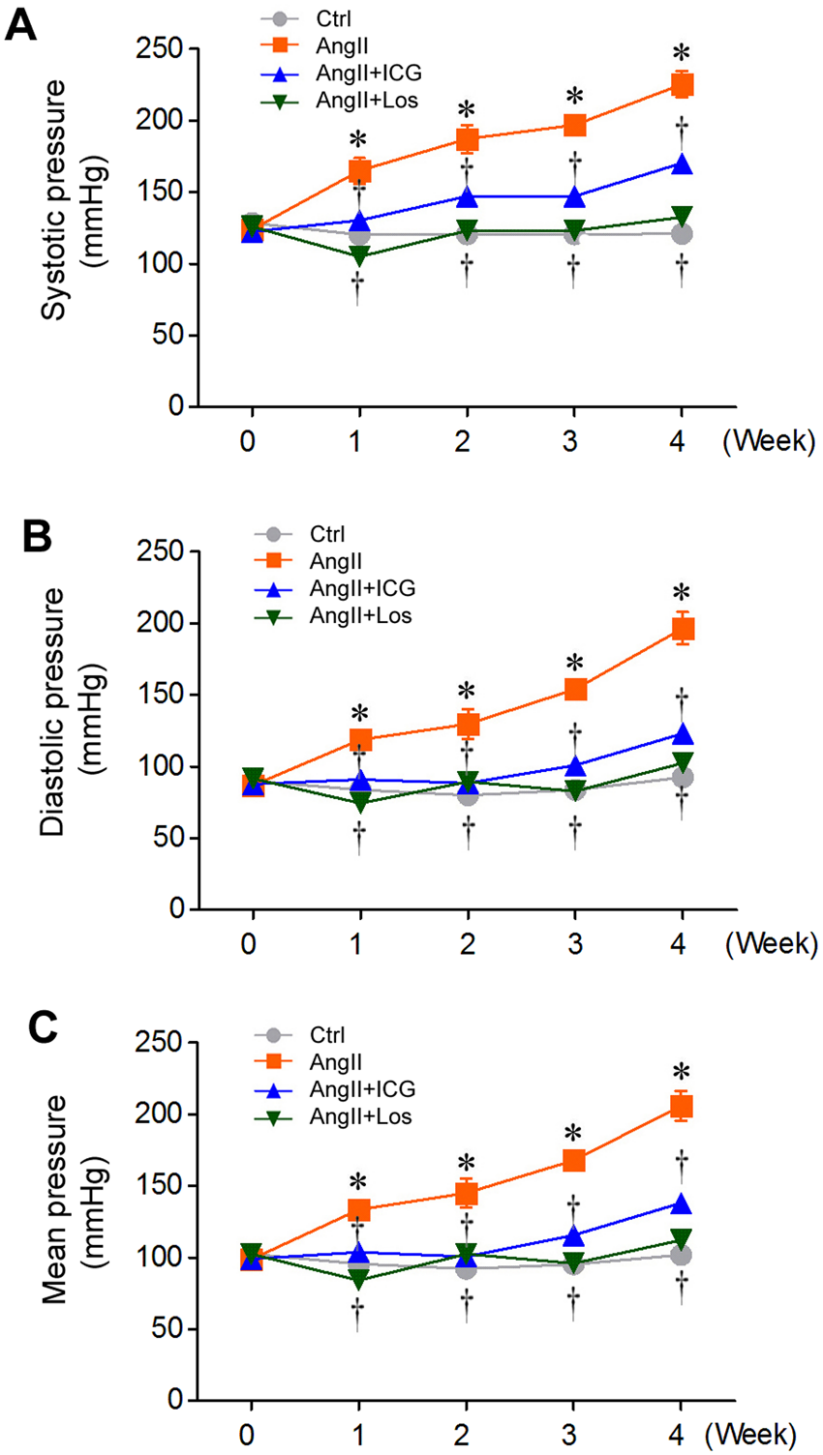
ICG-001 lowers blood pressure after Ang II infusion in rats. (**A**) Graphic presentation of the systolic arterial pressure of SD rats during 4 weeks in various treatment groups as indicated. (**B**) Graphic presentation of the diastolic arterial pressure of SD rats during 4 weeks. (**C**) Graphic presentation of the mean arterial pressure of SD rats during 4 weeks. Data are presented as mean ± SEM (n = 5). Ctrl, sham controls. ICG, ICG-001. Los, Losartan. *P < 0.05 versus controls; ^†^*P* < 0.05 versus Ang II alone.

Chronic Ang II infusion is well known to cause hypertension, in addition to inducing cardiac hypertrophy and fibrosis. Therefore, we assessed whether inhibition of β-catenin signaling by ICG-001 affects blood pressure. Figure 5 outlined distinct dynamic of blood pressure among different groups. Ang II infusion significantly induced hypertension, with elevated systolic blood pressure (SBP), diastolic blood pressure (DBP) and mean arterial pressure (MAP) in rats at different time points. As expected, losartan was able to normalize the blood pressure after Ang II infusion by virtue of its ability to block AT1 receptor (Fig. 5A through C). Interestingly, inhibition of β-catenin signaling by ICG-001 also significantly lowered blood pressure (Fig. 5A through C), although it appeared slightly less effective than losartan, particularly in later time points. These data suggest that Ang II-induced elevation in blood pressure is also dependent on, at least partially, the activation of Wnt/β-catenin signaling.

### Ang II stimulates Wnt/β-catenin activation in cardiomyocytes *in vitro*

**Figure 6:**
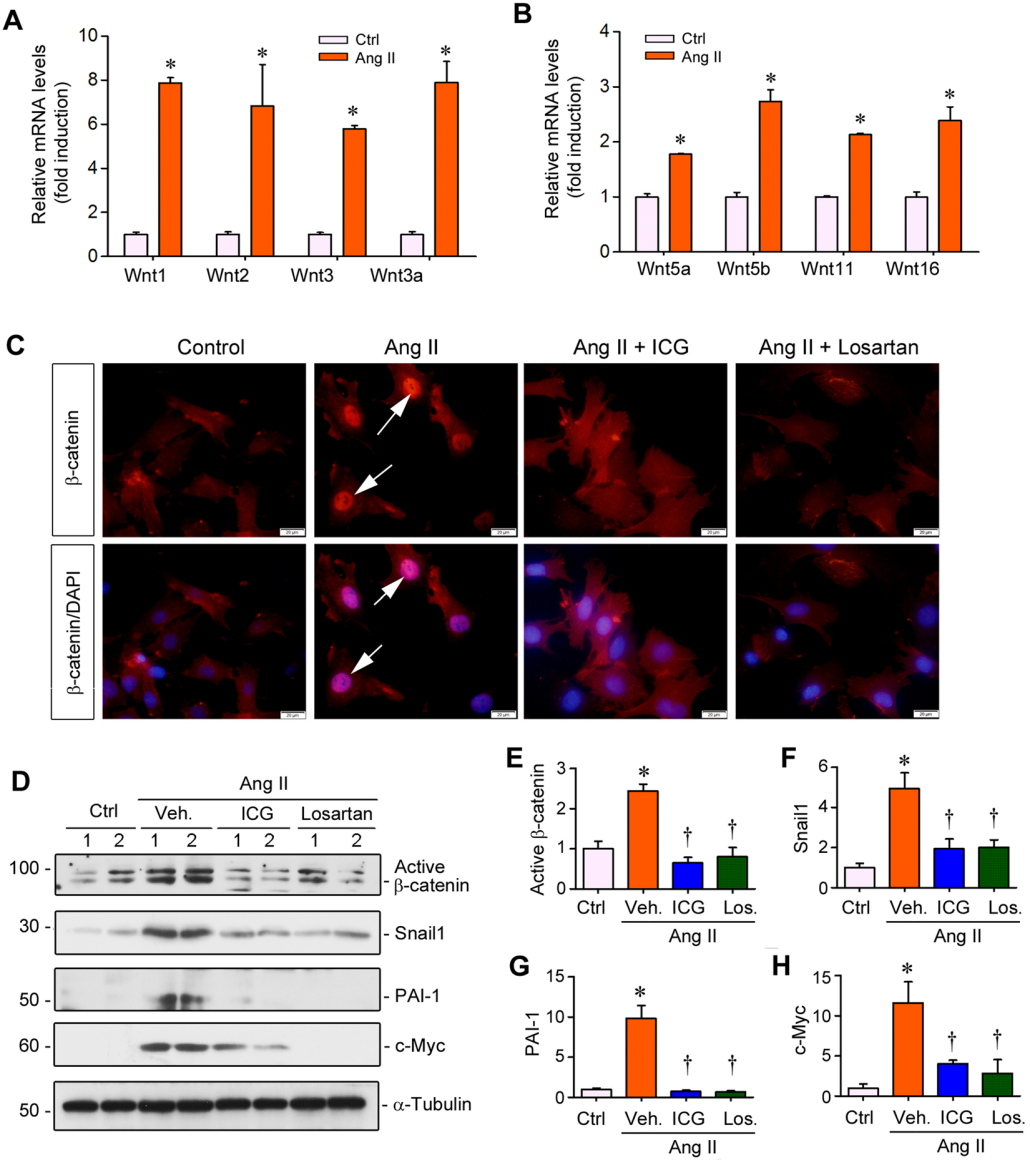
Ang II induces Wnt/β-catenin activation in primary cardiomyocytes. (**A**,**B**) qRT-PCR showed that a battery of Wnts mRNA was induced by Ang II. Primary cardiomyocytes were incubated with Ang II (10^−6^ M) for 12 hours. (**C**) Immunofluorescent staining for β-catenin in cardiomyocytes treated with Ang II in the absence or presence of ICG-001 or losartan. Arrows indicate nuclear localization of β-catenin after treatment with Ang II in primary cardiomyocytes. (**D**) Representative Western blot showed protein levels of active β-catenin, Snail1, PAI-1 and c-Myc in primary cardiomyocytes after various treatments for 24 hours as indicated. (**E**–**H**) Graphic presentation showed the relative protein levels of active β-catenin (**E**), Snail1 (**F**), PAI-1 (**G**) and c-Myc (**H**) in cardiomyocytes after various treatments. Ctrl, controls. Veh, Vehicle. ICG, ICG-001. Los, Losartan. *P < 0.05 versus controls; ^†^*P* < 0.05 versus Ang II alone (n = 5).

To validate the role of Wnt/β-catenin in mediating cardiac hypertrophy, we employed an *in vitro* system by using primary neonatal rat cardiomyocytes. Cardiomyocytes were isolated from the heart of neonatal rats and incubated with Ang II for 12 hours. As shown in Fig. 6A,B, qRT-PCR demonstrated that Ang II significantly induced the mRNA expression of multiple Wnt ligands in rat cardiomyocytes, including Wnt1, Wnt2, Wnt3, Wnt3a, Wnt5a, Wnt5b, Wnt11 and Wnt16 (Fig. 6A,B). Induction of Wnt ligands in cardiomyocytes could result in β-catenin activation in an autocrine fashion, which led to β-catenin stabilization and its nuclear translocation, as illustrated by immunofluorescent staining for β-catenin protein (Fig. 6C). Western blot analysis confirmed that Ang II not only induced β-catenin activation, but also promoted the expression of its downstream target genes such as Snail1, PAI-1 and c-Myc in cultured cardiomyocytes at 24 hours after incubation (Fig. 6D). Consistent with *in vivo* data, incubation of rat primary cardiomyocytes with ICG-001 or losartan blocked Ang II-induced β-catenin activation and its target gene expression (Fig. 6D through H).

### Inhibition of β-catenin signaling abolishes cardiomyocyte hypertrophy *in vitro*

**Figure 7:**
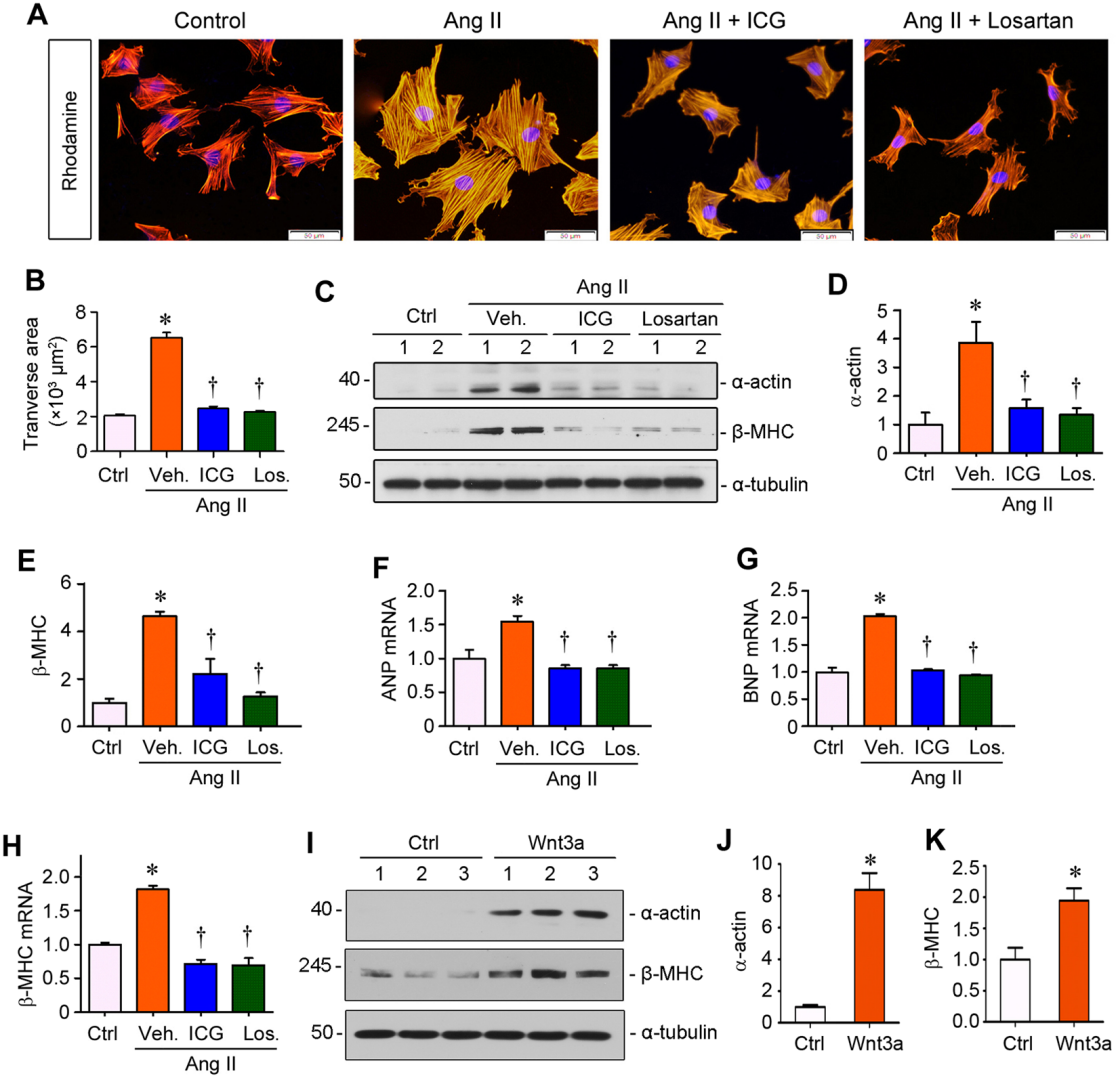
Inhibition of β-catenin by ICG-001 prevents Ang II-induced cardiomyocyte hypertrophy *in vitro*. (**A**) Representative micrographs showed rhodamine staining of cardiomyocytes stimulated by Ang II in the absence or presence of ICG-001 or losartan. Scale bar, 50 µm. (**B**) Graphic presentation showed the relative sizes of cardiomyocytes in various groups as indicated. *P < 0.05 versus controls; ^†^*P* < 0.05 versus Ang II alone (n = 15). (**C**) Representative Western blot analysis showed the protein levels of α-actin and β-MHC in cardiomyocytes after various treatments as indicated. (**D**,**E**) Graphic presentation showed the relative levels of α-actin and β-MHC proteins. *P < 0.05 versus controls; ^†^*P* < 0.05 versus Ang II alone (n = 5). (**F**–**H**) qRT-PCR demonstrated the mRNA levels of ANP, BNP and β-MHC in cardiomyocytes after various treatments as indicated. (**I**–**K**) Western blots showed that recombinant Wnt3a induced α-actin and β-MHC expression in primary cardiomyocytes. Rat cardiomyocytes were treated with Wnt3a (100 ng/ml) for 24 hours. Western blot (I) and quantitative data (**J** and **K**) are presented. Ctrl, controls. Veh, Vehicle. ICG, ICG-001. Los, Losartan. *P < 0.05 versus controls; ^†^*P* < 0.05 versus Ang II alone (n = 5).

We next examined the relationship between Wnt/β-catenin activation and cardiomyocyte hypertrophy *in vitro*. Rhodamine staining for actin cytoskeleton revealed that rat cardiomyocytes underwent hypertrophic change when exposed to Ang II, with an enlarged cell size (Fig. 7A). Both ICG-001 and losartan completely abolished the hypertrophic response of primary cardiomyocytes to Ang II stimulation (Fig. 7A,B). As shown in Fig. 7C through E, Western blot analysis revealed that ICG-001 and losartan markedly reduced the expression of α-actin and β-MHC in the presence of Ang II. Assessment of mRNA expression by qRT-PCR demonstrated that Ang II induced ANP, BNP and β-MHC expression in primary cardiomyocytes, and ICG-001 or losartan completely inhibited their expression (Fig. 7F through H). These data indicate that Ang II-induced cardiomyocyte hypertrophy and dysfunction is directly mediated by Wnt/β-catenin activation.

To further validate a role for Wnt signaling in mediating cardiac hypertrophy, we treated rat primary cardiomyocytes with recombinant Wnt3a. As shown in Fig. 7I through K, both α-actin and β-MHC were markedly induced in primary cardiomyocytes after incubation with Wnt3a for 24 hours. These results suggest that Wnt3a, which was induced in cardiomyocyes after Ang II stimulation (Fig. 1F and 6A), is sufficient to induce cardiomyocyte hypertrophy in an autocrine fashion.

### Wnt/β-catenin mediates Ang II-induced fibroblast activation

**Figure 8:**
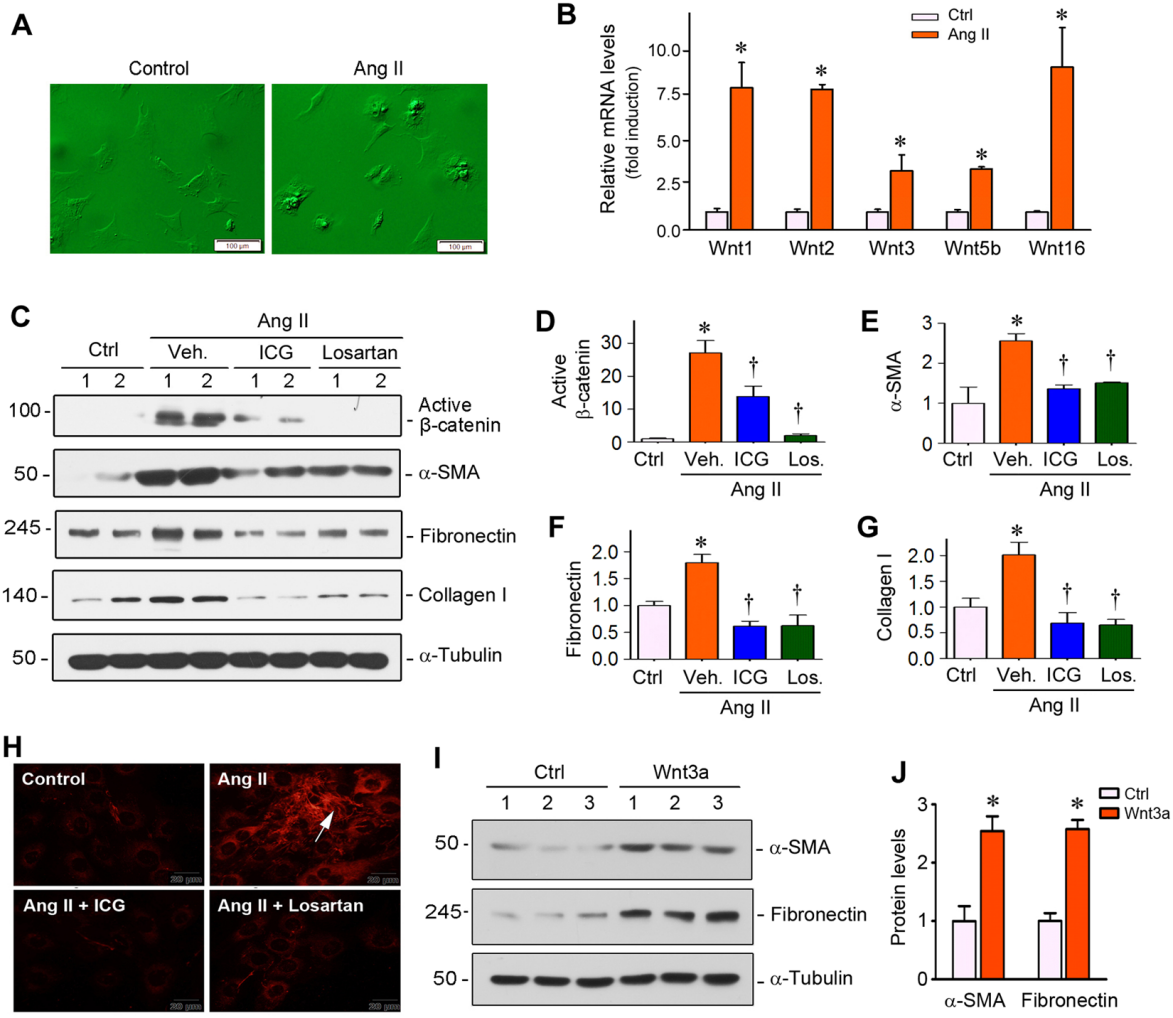
Inhibition of β-catenin by ICG-001 suppresses Ang II-induced fibroblast activation *in vitro*. (**A**) Representative phase-contrast micrographs of primary cardiac fibroblasts in the absence or presence of Ang II *in vitro*. Scale bar, 100 µm. (**B**) qRT-PCR showed that Ang II induced mRNA expression of a group of Wnt ligands in cultured cardiac fibroblasts. *P < 0.05 versus controls. (**C**) Representative Western blot showed the protein level of active β-catenin, α-SMA, fibronectin and collagen I in cardiac fibroblasts in various groups as indicated. (**D**–**G**) Graphic presentation showed the relative abundances of active β-catenin (**D**), α-SMA (**E**), fibronectin (**F**) and collagen I (**G**). *P < 0.05 versus controls; ^†^*P* < 0.05 versus angiotensin II alone (n = 5). (**H**) Immunofluorescent staining showed fibronectin protein deposition by fibroblasts after various treatments as indicated. Scale bar, 20 µm. Ctrl, controls. ICG, ICG-001. (**I**,**J**) Recombinant Wnt3a induced α-SMA and fibronectin expression in primary cardiac fibroblasts. Rat cardiac fibroblasts were treated with Wnt3a (100 ng/ml) for 24 hours. Western blot (**I**) and quantitative data (**J**) are presented.

We finally investigated the potential role of Wnt/β-catenin in mediating cardiac fibrosis by an *in vitro* system. As myofibroblastic activation of cardiac fibroblasts is a key event in cardiac fibrogenesis, we examined the regulation of this process *in vitro* by using primary culture of cardiac fibroblasts. As shown in Fig. 8A, primary cardiac fibroblasts were isolated from neonatal rats and incubated in the absence or presence of Ang II. We then tested whether Ang II regulates Wnt expression by qRT-PCR in cultured fibroblasts. As shown in Fig. 8B, multiple Wnt ligands were induced by Ang II, including Wnt1, Wnt2, Wnt3, Wnt5b and Wnt16 in cardiac fibroblasts. As a result of these Wnts induction, β-catenin was markedly activated, as demonstrated by Western blot analysis using specific antibody against active β-catenin (Fig. 8C,D). Concomitant with Wnt/β-catenin activation, Ang II also induced α-SMA expression and upregulated matrix proteins such as fibronectin and collagen I (Fig. 8C through G). Treatment with either ICG-001 or losartan dramatically inhibited Ang II-induced β-catenin activation, as well as α-SMA, fibronectin and collagen I expression in cardiac fibroblasts (Fig. 8C through G). Similar results were obtained when fibronectin deposition was assessed by immunofluorescence staining (Fig. 8H).

When cardiac fibroblasts were incubated with Wnt3a, both α-SMA and fibronectin were induced (Fig. 8I,J), suggesting that Wnt ligands are sufficient to cause cardiac fibroblast activation as well, leading to matrix over-production. Therefore, it is concluded that Wnt/β-catenin also plays an essential role in mediating Ang II-induced cardiac fibroblast activation and matrix production.

## Discussion

**Figure 9:**
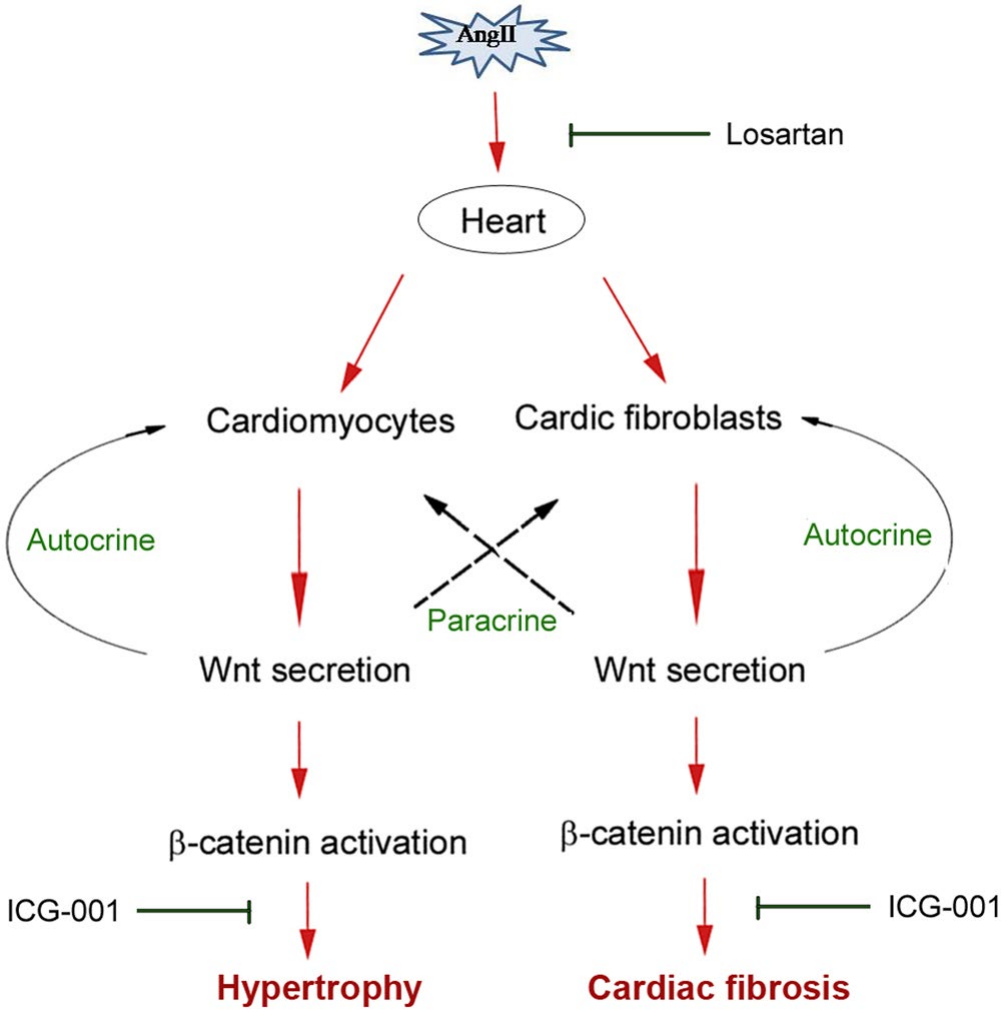
Schematic diagram depicts the role of Wnt/β-catenin signaling in mediating Ang II-induced cardiac hypertrophy and fibrosis. Ang II induces *de novo* expression of a variety of Wnt ligands in both cardiomyocytes and cardiac fibroblasts, and then stimulates β-catenin activation by autocrine and paracrine mechanism. As expected, blockade of Ang II action by losartan represses Wnt/β-catenin signaling. Similarly, inhibition of β-catenin signaling by ICG-001 also blocks cardiomyocyte hypertrophy and fibroblast activation, thereby preventing Ang II-induced hypertension, cardiac injury, hypertrophy and fibrosis.

In the present study, we have provided multiple lines of evidence suggesting an essential role of Wnt/β-catenin in mediating cardiac hypertrophy, myocardial fibrosis and hypertension in rats. Using a classic model of hypertensive heart disease induced by chronic infusion of Ang II, which imitates systemic RAS activation, we show that canonic Wnt signaling is indispensable in the development of cardiac lesions and hypertension. As summarized in Fig. 9, Ang II induces multiple Wnt ligands in both cardiomyocytes and cardiac fibroblasts *in vivo* and *in vitro*. As a result, β-catenin is activated in these cells presumably via both autocrine and paracrine mechanisms. Activation of β-catenin in cardiomyocytes induces hypertrophic markers β-MHC and α-actin, activates c-Myc and Snail1 transcription factors, and stimulates ANP and BNP expression. Such changes ultimately lead to myocyte hypertrophy and cardiac dysfunction. Meanwhile, β-catenin activation in cardiac fibroblasts causes their myofibroblastic transformation, thereby enhancing their ability to produce and secrete interstitial matrix components such as fibronectin and collagen I, leading to myocardial fibrosis (Fig. 9). These studies provide novel insights into the mechanism by which Ang II causes cardiac hypertrophy, fibrosis and dysfunction. Our findings may also have a broad implication in defining the mediators governing cardiac hypertrophy, the common response of the heart to diverse insults in a wide variety of cardiovascular disorders.

One interesting finding in this study is the simultaneous induction of multiple Wnt ligands in the heart after chronic infusion of Ang II. Wnt ligands are a family of glycoproteins that contains 19 distinct members^16,17,28^. Although β-catenin has been implicated in regulating Ang II-triggered myocyte hypertrophy *in vitro*^21,33,34^, little was known previously about Wnt regulation in the hypertrophic heart *in vivo*. After a comprehensive analysis of the mRNA expression of all 19 Wnt ligands, we found that 8 of them were substantially induced in the hypertrophic heart after Ang II infusion in rats (Fig. 1). This result is further corroborated in primary rat cardiomyocytes *in vitro*, in which many of the same Wnt ligands including Wnt1, Wnt2, Wnt3, Wnt3a, Wnt5a and Wnt5b were induced by Ang II (Fig. 6). It should be pointed out that we did not observe a significant down-regulation of any Wnt ligands either in the hypertrophic heart or in cultured cardiomyocytes after Ang II treatment (data not shown), suggesting that induction of Wnt expression is an overwhelmingly predominant response after Ang II stimulation. Not surprisingly, such a concomitant upregulation of multiple Wnt ligands subsequently leads to marked stabilization and activation of β-catenin, as illustrated by an increased expression of both total and active β-catenin protein, as well as its downstream targets (Fig. 1). Taken together, these results underscore that Ang II, the principal effector of the RAS^35,36^, is a master upstream regulator that controls the expression of many Wnt ligands in the heart.

The connection between RAS and Wnt/β-catenin might not be a one-way street. Rather, it appears to be bidirectional and reciprocal. While Ang II stimulates the expression of multiple Wnt ligands (Fig. 1), our previous studies demonstrate that Wnt/β-catenin also induces multiple components of the RAS, including angiotensinogen, renin, angiotensin-converting enzyme, and AT1 receptor^37,38^. Therefore, RAS activation and Wnt/β-catenin could form a vicious cycle, leading to a dramatic amplification of this powerful signal loop and promoting myocyte hypertrophy and dysfunction. The mechanism by which Wnt/β-catenin promotes cardiac hypertrophy was largely elusive, but the present study suggests that β-catenin could directly control the expression of the hypertrophic marker genes. This conclusion is supported by the observation that recombinant Wnt3a induces both α-actin and β-MHC expression in primary cardiomyocytes (Fig. 7), illustrating that Wnt is sufficient to cause hypertrophic response of cardiomyocytes. Furthermore, ICG-001 abolishes α-actin and β-MHC induction in cultured cardiomyocytes (Fig. 7). Wnt/β-catenin also induces fetal genes such as ANP and BNP, which are widely recognized as markers of heart damage^4^. In agreement with this notion, inhibition of Wnt/β-catenin by ICG-001 mitigates a multitude of geometric cardiac changes in rats after Ang II infusion (Fig. 3), highlighting a role of canonic Wnt signaling in regulating cardiac function.

Our studies also pinpoint cardiac fibroblasts as a crucial player in the pathogenesis of hypertensive heart disease. Several Wnt ligands are induced by Ang II in primary rat cardiac fibroblasts *in vitro* (Fig. 8), and Wnt3a, which is induced in cardiomyocytes after Ang II stimulation (Fig. 6), causes cardiac fibroblast activation presumably by a paracrine fashion (Fig. 8), suggesting that fibroblasts are both Wnt-producing cells and Wnt-responding cells, via both autocrine and paracrine action (Fig. 9). Notably, Wnt expression profile in cardiomyocytes and cardiac fibroblasts after Ang II incubation is not exactly the same, suggesting the cell type-specificity of Wnt expression in response to Ang II. The crucial role for Wnt/β-catenin in cardiac fibroblast activation is supported by the observation that Wnt3a induces α-SMA and fibronectin expression, whereas ICG-001 abolishes their induction by Ang II (Fig. 8). These findings are in line with numerous earlier reports indicating a role of Wnt/β-catenin in mediating fibroblast activation in many organs including the heart and kidney^29,39–41^.

Another novel finding in the present study is that blockade of Wnt/β-catenin by ICG-001 lowers blood pressure after Ang II infusion (Fig. 5), suggesting that this signal pathway is a critical player previously unrecognized in the blood pressure regulation. The ability of ICG-001 to lower blood pressure is comparable to losartan, an AT1 receptor blocker that obliterates Ang II-triggered hypertension by virtue of its ability to block Ang II action in the first place. The underlying mechanism by which ICG-001 abolishes Ang II-induced elevation of blood pressure in rats remains largely unknown at this stage. However, one possibility could be related to its ability to inhibit the induction of endogenous RAS genes^37^. Since Ang II-induced hypertension requires *de novo* activation of RAS, its blockade by ICG-001 would disrupt the vicious cycle of exogenous Ang II infusion/Wnt/β-catenin activation/endogenous RAS induction.

The present study has some limitations. In particular, ICG-001 was administered concomitantly with Ang II infusion, which raises the possibility that cardiac protection by ICG-001 may be a result of the normalization of blood pressure. However, administration of Ang II is continuous and chronic process using the mini-pump approach; and Ang II can cause cardiac injury by a mechanism independent of the elevated blood pressure. Furthermore, recombinant Wnt3a is sufficient to induce cardiomyocyte injury and fibroblast activation (Figs 7 and 8). Given that ICG-001 inhibits Ang II-induced cardiomyocyte hypertrophy and fibroblast activation both *in vivo* and *in vitro*, it is conceivable that activation of Wnt/β-catenin could play an essential role in mediating cardiac damage. Future studies are needed to test whether ICG-001 can reverse or mitigate an established cardiac pathology.

In summary, we have shown herein that Ang II induces cardiac expression of multiple Wnt ligands and activates β-catenin both *in vivo* and *in vitro*, and Wnt3a is sufficient to cause cardiomyocyte injury and fibroblast activation. Blockade of this signal cascade by small molecule inhibitor ICG-001 is cardiac protective by inhibiting myocyte hypertrophy and fibrosis, and lowering blood pressure. Although more studies are needed, our findings suggest that Wnt/β-catenin could be a novel target for the treatment of hypertensive heart diseases.

## Methods

### Animal Models

Male Sprague Dawley (SD) rats, weighing approximately 200–220 g, were purchased from the Experimental Animal Center of the Southern Medical University (Guangzhou, China). SD rats were randomly divided into four groups (n = 5): (1) sham controls chronically infused with PBS; (2) model chronically infused with Ang II (Bachem, Torrance, CA) at the rate of 450 ng/min/kg via osmotic pumps (Alzet Model 2004, DURECT Corp. Cupertino, CA); (3) Ang II infusion rats treated with ICG-001 (847591-62-2; Chembest, Shanghai, China) at dosage of 5 mg/kg body wt daily by intraperitoneal injection; (4) Ang II infusion rats given oral Losartan (Merck sharp & Dohme, Kenilworth, NJ) at dosage of 10 mg/kg body wt daily. For osmotic mini-pump implantation, SD rats were anesthetized by pentobarbital sodium, shaved and sterilized on mid-scapular region. A subcutaneous pocket was made for mini-pump implantation, followed by suturing incision. At 4 weeks after osmotic pumps implantation, rats were euthanized and sacrificed. Heart tissues and blood were collected for various analyses. All animal studies were performed in accordance with US Public Health Service policy on humane care and use of laboratory animals, and approved by the Animal Ethics Committee at the Nanfang Hospital, Southern Medical University.

### Cell Culture

Primary neonatal rat ventricular cardiomyocytes (NRVCs) were isolated and cultivated for this study, according to the protocols previously described^42^. Briefly, after euthanasia by 2% isoflurane inhalation and cervical dislocation, heart was removed from SD neonatal rats of 3 days after birth. Cardiomyocytes and cardiac fibroblast were isolated by enzymes digestion including tryptase and collagenase II, as reported elsewhere^42^. Primary cells were cultured in DMEM/F12 medium with 10% fetal bovine serum under standard condition. BrdU was added into culture medium at final concentration of 0.1 mM to inhibit fibroblast proliferation in cardiomyocytes. After 48 h incubation in complete medium, cells were starved in serum-free medium overnight, and then followed by various treatments. These primary cardiomyocytes exhibited spontaneous beating on the dish. The purity of primary cardiomyocyte population was more than 95% based on the morphology and the staining for α-actin (data not shown). Primary cells were treated with Ang II (1 µM) in the absence or presence of ICG-001 (5 µM) and losartan (10 µM; Sigma-Aldrich, St. Louis, MO). Primary cardiomyocytes were also treated with recombinant Wnt3a (5036-WN; R&D Systems, Minneapolis, MN). At different time points after treatment, cells were harvested for total RNA extraction and protein isolation, respectively, or subjected to various analyses.

Primary cardiac fibroblasts were isolated from the hearts of neonatal rats. Briefly, rats in 3-day age were sacrificed by 2% isoflurane inhalation and cervical dislocation. Hearts were incised and rinsed with PBS precooled in 4 °C. Then, hearts were pre-digested in 4 °C with 0.25% trypsin for 12 hours, followed by incubation with 1 mg/ml collagenase II to make cell suspension. Isolated cells were seeded in culture plate and incubated for 90 minutes at 37 °C in CO_2_ incubator. Adherent cells were cultivated in DMEM/F12 medium with 10% fetal bovine serum. Cardiac fibroblasts within 3 passages were subjected to various treatments as indicated.

### Western Blot Analysis

Total protein from heart tissue and cultured cells was lysed in buffer containing 1% NP40, 0.1% SDS, 1 mg/ml PMSF, 1% protease inhibitor cocktail, and 1% phosphatase I and II inhibitor cocktail (Sigma). Western blot analysis for specific protein expression was carried out according to standard procedures, as reported previously^39^. The primary antibodies used were as follows: β-MHC (MAB90961; R&D systems), α-actin (KM9006T; Sungene Biotech, Tianjin, China), β-catenin (610154; BD biosciences, San Jose, CA), active β-catenin (05-665; EMD Millipore, Billerica, MA), PAI-1 (AF1786; R&D systems, Minneapolis, MN), Snail1 (ab180714; Abcam, Cambridge, UK), fibronectin (F3648; Sigma, Darmstadt, Germany), α-SMA (clone 1A4; Sigma, Darmstadt, Germany), collagen I (BA0325; Boster, Pleasanton, CA), c-Myc (D84C12; CST, Massachusetts, USA) and α-tubulin (RM2007; Ray antibody Biotech, Beijing, China). Relative protein abundance was calculated after normalizing with α-tubulin.

### qRT-PCR

Total RNA was extracted from heart tissues or cultured cells with TRIzol reagent according to the instruction of the manufacturer (Life Technologies, Grand Island, NY). The first strand cDNA was synthesized using 2 µg RNA in 20 µl of reaction system by a Reverse Transcription System kit (Promega, Madison, Wisconsin). Quantitative, real-time RT-PCR (qRT-PCR) was performed on an ABI PRISM 7000 Sequence Detection System (Applied Biosystems, Foster City, CA), as described elsewhere^43^. PCR reaction system contained 12.5 µl SYBR Green PCR MasterMix (Applied Biosystems), 5 µl diluted RT product (1:10), and 0.5 mM sense and antisense primer sets. The primer pairs for qRT-PCR are presented in Supplemental Table 1. In brief, PCR protocol was composed of incubations at 50 °C for 2 min, 95 °C for 10 min, and the amplification reaction consisted of 40 cycles including denaturing at 95 °C for 15 seconds, annealing and extension at 60 °C for 60 seconds. Relative mRNA levels were reported after normalization with β-actin.

### Histology and Immunohistochemical Staining

Paraffin sections were prepared by routine procedures. The sections were stained with hematoxylin-eosin (H.E.) according to standard protocol. Heart tissue sections were also subjected to Masson’s trichrome staining (MTS) for assessing fibrotic lesions. Immunohistochemical staining was carried out by using standard protocol^39^. The primary antibodies were as follows: rabbit polyclonal anti-β-catenin antibody (ab15180; Abcam), Wnt3a (SAB2108434; Sigma, Darmstadt, Germany), β-MHC (sc-53089; Santa Cruz, CA), α-actin (KM9006; Sungene, Tianjin, China), fibronectin (F3648; Sigma, Darmstadt, Germany). Micrographs were captured on Leica fluorescence microscope.

### Immunofluorescence Staining

Cardiomyocytes or cardiac fibroblasts seeded on coverslips were fixed with 3.7% paraformalin buffer for 15 min at room temperature, and then permeabilized with 0.5% Triton X- 100 for 10 min, followed by blocking with 10% donkey serum for 30 min. The slides were then incubated with primary antibodies specifically against β-catenin (15B8; Thermo Fisher, Rockford, IL) and fibronectin (F3648; Sigma, Darmstadt, Germany) as indicated. To visualize the primary antibodies, slides were stained with cyanine dye-2 or cyanine dye-3–conjugated secondary antibodies (Jackson Immuno-Research Laboratories, West Grove, PA). Some cells were also stained with Rhodamine or DAPI to visualize actin and nuclei, respectively. Slides were viewed under Leica fluorescence microscope equipped with a digital camera.

### Echocardiography

Cardiac function was assessed by Doppler echocardiography (VisualSonics Vevo2100 Imaging system, Toronto, Ontario, Canada) with a 21-MHz transducer (MS250). Rats were mild anesthetized by inhalant 3.0% isoflurane and oxygen at rate of 1 L/min. Images were standardized to short axis view at the LV mid-papillary level. Two-dimensional image for 3 sequential cardiac cycles were recorded. Posterior wall diastolic thickness (LVPWd), LV end-diastolic diameter (LVEDd) and LV end-systolic diameter (LVESd) were measured. LV systolic function parameters such as the LV fractional shortening (LVFS) and the LV ejection fraction (LVEF) were calculated.

### Blood Pressure Measurement

Tail-cuff measurements of systolic (SBP) and diastolic blood pressures (DBP) were performed by a validated method that relies on volume pressure recording (VPR) technology (CODA 8; Kent Scientific Corporation, Torrington, CT)^43^. This method has been validated by using a radio-telemetry system^44–46^. In brief, before measurement, SD rats were fixed comfortably and kept at rest in tube-shaped holder. Room temperature was set at 26 °C. On the Kent warming panel, blood pressure measuring was repeated for 9 times upon temperature at base of tail reached 32 °C. Normal measuring process was screened out by blood measuring software system. Average blood pressure of repeated measurements was reported.

### BNP measurement by ELISA

Blood was collected and kept for 2 hours in room temperature. After coagulation, blood sample was centrifuged with 3000 rpm, 30 min at 4 °C. Supernatant was collected and stored in −80 °C. Serum BNP was measured using ELISA kit for BNP according to the protocols specified by the manufacturer (CEA541Ra; USCN life Science Inc., Wuhan, China). The results were expressed as pg per ml.

### Statistical analyses

All data examined were expressed as mean ± SEM. Statistical analysis of the data was carried out using GraphPad Prism software (La Jolla, CA). Comparison between groups was made using one-way ANOVA followed by Student-Newman-Kuels test. *P* < 0.05 was considered statistical significance.

## Electronic supplementary material

Supplementary Table 1
