# Supplementary material for: An essential role for Wnt/β-catenin signaling in mediating hypertensive heart disease
Source: Sci Rep. 2018 Jun 12;8:8996. doi: 10.1038/s41598-018-27064-2 (PMC5997634; doi:10.1038/s41598-018-27064-2)
Supplement: Supplementary file 1 — Supplementary Table 1 [file 41598_2018_27064_MOESM1_ESM.doc]

**An essential role for Wnt/β-catenin signaling in mediating hypertensive heart disease**

Yue Zhao,^1^ Chunhong Wang,^1^ Cong Wang,^1^ Xue Hong,^1^ Jinhua Miao,^1^ Yulin Liao,^2^ Lili Zhou,^1^ Youhua Liu^1,3*^

^1^State Key Laboratory of Organ Failure Research, National Clinical Research Center of Kidney Disease, Division of Nephrology, ^2^Division of Cardiology, Nanfang Hospital, Southern Medical University, Guangzhou, China; ^3^Department of Pathology, University of Pittsburgh School of Medicine, Pittsburgh, Pennsylvania

Running title: Wnt and cardiac hypertrophy

*To whom correspondence should be addressed:

Youhua Liu, Ph.D, Department of Pathology, University of Pittsburgh School of Medicine, S-405 Biomedical Science Tower, 200 Lothrop Street, Pittsburgh, PA 15261. E-mail: [yhliu@pitt.edu](mailto:yhliu@pitt.edu).

| Genes | Forward Primer | Reverse Primer |
| --- | --- | --- |
| Wnt1 | 5'- GCCCTAGCTGCCAACAGTAGT -3' | 5'- GAAGATGAACGCTGTTTCTCG -3' |
| Wnt2 | 5'- AGAGTGCCAACACCAGTTCC -3' | 5'- TACAGGAGCCACTCACACCA -3' |
| Wnt2b | 5'- TTGTGTCAACGCTACCCAGA -3' | 5'- ACCACTCCTGCTGACGAGAT -3' |
| Wnt3 | 5'- GGGGCGTATTCAAGTAGCTG -3' | 5'- GTAGGGACCTCCCATTGGAT -3' |
| Wnt3a | 5'- TTCTTACTTGAGGGCGGAGA -3' | 5'- CTGTCGGGTCAAGAGAGGAG -3' |
| Wnt5a | 5'- CCCAGTCCGGACTACTGTGT -3' | 5'- TTTGACATAGCAGCACCAGTG -3' |
| Wnt5b | 5'- TCTCCGCCTCACAAAAGTCT -3' | 5'- CACAGACACTCTCAAGCCCA -3' |
| Wnt9a | 5'- CCCCTGACTATCCTCCCTCT -3' | 5'- GATGGCGTAGAGGAAAGCAG -3' |
| Wnt11 | 5'- TGCTTGACCTGGAGAGAGGT -3' | 5'- AGCCCGTAGCTGAGGTTGT -3' |
| Wnt16 | 5'- CCCTCTTTGGCTATGAGCTG -3' | 5'- TACTGGACATCATCCGAGCA -3' |
| ANP | 5'- CGAGGTGACAGAGACCACAA -3' | 5'- CTGGAGTCAAGCCAGACACA -3' |
| BNP | 5'- GAGGCACCACTGAACCCTAA -3' | 5'- CATCTCCAGAGTCCAGCACA -3' |
| β-MHC | 5'- ATCTCCTGGTGCTGATGGAC -3' | 5'- ACCTTGTTTGCCAGGTTCAC -3' |

**Table S1.** Nucleotide sequences of the primer pairs used for qRT-PCR
